# Phylogenetic diversification and fitness trade-offs of TetA variants in mediating eravacycline resistance in *Klebsiella pneumoniae*

**DOI:** 10.1128/aac.00671-25

**Published:** 2025-12-30

**Authors:** Meimei Fan, Zhixun Zhang, Xiaowei Liu, Liqin Deng, Yijie Lei, Feng Chen, Yuanyuan Ying, Chengfeng Fan, Jiaxin Gao, Zheer Ren, Jiayi Gu, Yuying Xia, Yifan Yuan, Keyu Zhang, Junfei Zhu, Yilin Yang, Weining Sun, Kaiying Cheng, Alessandra Carattoli, Christoph M. Tang, Zhen Shen, Guangyu Liu

**Affiliations:** 1 Zhejiang Key Laboratory of Medical Epigenetics, Department of Immunology and Pathogen Biology, School of Basic Biomedical Sciences, Hangzhou Normal University 26494, Hangzhou, Zhejiang, China; 2 Department of Molecular Medicine, Sapienza University of Rome, Rome, Italy; 3 Sir William Dunn School of Pathology, University of Oxford 6396, Oxford, United Kingdom; 4 Department of Laboratory Medicine, Renji Hospital, School of Medicine, Shanghai Jiao Tong University, Shanghai, China; University of Fribourg, Fribourg, Switzerland

**Keywords:** advanced tetracyclines, plasmids, fitness costs, heavy metals, disinfectants, ecological trade-off

## Abstract

The role of TetA variants in mediating tigecycline and eravacycline resistance in *Klebsiella pneumoniae* remains a critical area of investigation. However, there has been a lack of systematic characterization of the epidemiology, resistance phenotypes, and fitness costs of TetA variants. Here, we identified 28 TetA variants in *K. pneumoniae* from the National Center for Biotechnology Information database from 824 isolates, categorizing them into three phylogenetically distinct clades. Among these, four variants were shown to mediate eravacycline resistance, with concurrent but variable effects on tigecycline susceptibility. Notably, these resistance-conferring variants exhibited limited dissemination across clinical and environmental strains. Analyses revealed that their expression imposes a significant fitness cost, markedly reducing bacterial tolerance to the clinical disinfectant H_2_O_2_ and an environmental heavy metal cadmium—a trait critical for survival under ecological stress. This trade-off likely explains the limited prevalence of these variants despite their resistance phenotypes. Our findings highlight the evolutionary constraints affecting the spread of TetA-mediated antibiotic resistance and underscore the need for One Health-driven surveillance to monitor variants with potential risk in human, animal, and environmental reservoirs. This work provides novel insights into the interplay between resistance determinants and bacterial adaptability, offering a framework for predicting resistance dynamics in *K. pneumoniae* within the context of interconnected ecological and clinical ecosystems.

## INTRODUCTION

Carbapenem-resistant *Klebsiella pneumoniae* (CRKP) poses a critical global health threat due to its propensity for multidrug resistance and nosocomial transmission (1, 2). While advanced tetracyclines such as tigecycline and eravacycline serve as last-line therapies against CRKP (3, 4), their escalating clinical use has precipitated a concerning rise in resistance (5–8).

Mechanistically, resistance to advanced tetracyclines primarily arises from enzymatic inactivation by TetX-family oxidases or drug extrusion via efflux pumps such as TMexCD-TOprJ, OqxAB, and MacAB (9–12). Notably, these determinants are frequently environmental or zoonotic in origin and remain underrepresented in clinical isolates, suggesting niche-specific barriers to their human adaptation (13, 14). In stark contrast, variants of TetA—a major facilitator superfamily (MFS) efflux pump—have emerged as predominant clinical drivers of tigecycline resistance in *K. pneumoniae* (15–17). Despite their clinical relevance, the evolutionary trajectories and ecological constraints governing TetA variant dissemination across the One Health continuum remain enigmatic.

Here, we bridge this gap through a systematic phylogenetic and functional dissection of TetA variants in *K. pneumoniae*. By mining National Center for Biotechnology Information (NCBI) genomes, we identified 28 TetA variants, 4 of which conferred eravacycline resistance, a previously unreported phenotype for this efflux pump family. Surprisingly, molecular epidemiology revealed limited spread of these variants with potential risk (VWPRs), hinting at uncharacterized fitness costs offsetting their resistance advantage.

Guided by the One Health paradigm, we hypothesized that the environmental stressors commonly present in hospital and ecological settings might impose selective barriers limiting the spread of VWPRs (18, 19). Consistent with this, *K. pneumoniae* expressing the TetA VWPRs exhibited hypersusceptibility to H_2_O_2_ (a common disinfectant) and cadmium. These findings implicate environmental heavy metals as not merely passive contaminants but also as key players modulating the dissemination of resistance genes—a dynamic overlooked in current surveillance frameworks.

## MATERIALS AND METHODS

### Epidemiological and phylogenetic analysis of *tetA* variants

The *tet*(A)v1 allele (GenBank Accession number X61367) was used as a query in BLASTn searches against the NCBI *K. pneumoniae* genome database (cutoff: ≥90% identity). Metadata (isolation source, geographic origin, and collection year) were curated for 28 non-redundant *tetA* homologs (Table S1). Amino acid sequences of TetA variants (Table S2) were aligned with Clustal Omega (20), visualized via iTOL (https://itol.embl.de). Variants were divided into clades based on phylogeny, and within each clade, variants were designated based on allele frequency. Transmembrane domain prediction was based on TetA-1.1 structure predicted by AlphaFold3 (21).

### Bacterial strains and culture conditions

All bacterial strains used in this study are listed in Table S3. Cloning host: *Escherichia coli* DH5α was cultivated in Lysogeny Broth (LB) medium at 37°C with agitation (150 rpm). *K. pneumoniae* strains—including clinical isolate Kp36 (22), TU37, HS11286 (23), and their derivatives—were grown under identical conditions. Antibiotics were supplemented as follows: carbenicillin (100 mg/L) for plasmid selection and kanamycin (50 mg/L) for mutant screening.

### Plasmid construction and molecular cloning

The tetracycline resistance gene *tetA-1.1* was originally from *K. pneumoniae* Kp36, cloned into a low-copy vector pUA139 (modified from pUA139 ada) (24). Gene fragments and vector backbones were amplified by PCR (Phanta Flash DNA Polymerase, Vazyme), assembled *in vitro* using Gibson Assembly Master Mix (ABclonal #RK21022), and transformed into *E. coli* DH5α via heat shock. Plasmid integrity was verified by Sanger sequencing (Tsingke Biotech), and validated constructs were purified (QIAprep Spin Miniprep Kit) for downstream applications. All plasmids and primers used in this study are listed in Tables S4 and S5, respectively.

### Antimicrobial susceptibility profiling

Minimum inhibitory concentrations (MICs) for tigecycline and eravacycline were determined by broth microdilution (Clinical and Laboratory Standard Institute M07-A11) (25) in cation-adjusted Mueller-Hinton broth. *K. pneumoniae* TU37 harboring *tetA* variants was tested in triplicate. Susceptibility interpretations followed the US Food and Drug Administration (https://www.fda.gov/drugs/development-resources/tigecycline-injection-products) (tigecycline: susceptible ≤2 mg/L, intermediate 4 mg/L, and resistant ≥8 mg/L) and China ECAST (eravacycline: susceptible ≤1 mg/L) criteria (26).

### Growth assays

Bacterial growth was measured in a real-time microbial growth curve system (Viewkr Biotechnology). Bacteria diluted to 6 × 10³ CFU/mL and the negative control (phosphate-buffered saline) were transferred to a 96-well plate at *T*_0_, which was then grown in LB, acidic LB (pH = 5.0), or M9 minimal media supplemented with anhydrotetracycline (aTC) (50 μg/L) as an inducer at 37°C. Bacterial growth data were acquired every 10 min for 35 h (*n* = 3). Before each measurement, the 96-well plate was agitated for 15 s at 30 Hz.

### Heavy metals and disinfectants susceptibility assays

In the initial screen, tetracycline (10 mg/L, 30 min)-induced *tetA*-expressing strains (HS11286 and TU37) were adjusted to OD_600_ = 0.1, plated on LB agar with tetracycline (10 mg/L), and exposed to 6-mm filter discs impregnated with heavy metals (K_2_TeO_3_ [0.1 M], CuSO_4_ [0.1 M], AgNO_3_ [0.1 M], CdCl_2_ [0.1 M], and NiCl_2_ [0.01 M]) and disinfectants (benzalkonium chloride [neat], sodium hypochlorite [10% dilution], and H_2_O_2_ [3% vol/vol]). Inhibition zones were measured after 16-h incubation at 37°C (*n* = 3). Disinfectants and heavy metals exhibit differential inhibitory effects on TetA VWPRs compared to TetA-1.1 and were tested again following the same protocol except tetracycline was replaced by aTC (50 μg/L) as the inducer.

### Time-kill assays

Overnight cultures of *K. pneumoniae* expressing TetA variants grown in LB supplemented with aTC (50 μg/L) and kanamycin (50 mg/L) were diluted and subcultured into 20 mL of LB supplemented with aTC (50 μg/L) and kanamycin (50 mg/L) with initial OD_600_ of 0.008, grown at 37°C with shaking (220 rpm) for 3 h before H_2_O_2_ or CdCl_2_ was added. Viable bacteria were measured by serial dilution and plating onto LB agar at 0, 2, 4, and 6 h post-addition of H_2_O_2_ or CdCl_2_. Final concentration of H_2_O_2_ used for killing is 0.012% (vol/vol) for both *K. pneumoniae* TU37 and HS11286. Final concentration of CdCl_2_ used for killing is 40 mM for *K. pneumoniae* TU37 and 2.5 mM for HS11286.

### Statistical analysis

Data were analyzed in GraphPad Prism 9.0. One-way or two-way analysis of variance (Tukey’s post hoc) was used to assess differences between groups in the disc diffusion susceptibility assays or time-kill assays, respectively. Significance thresholds: *P < 0.05, **P < 0.01, ***P < 0.001, ****P < 0.0001.

## RESULTS

### Global dissemination and clinical shifts in TPKP isolates

**Fig 1:**
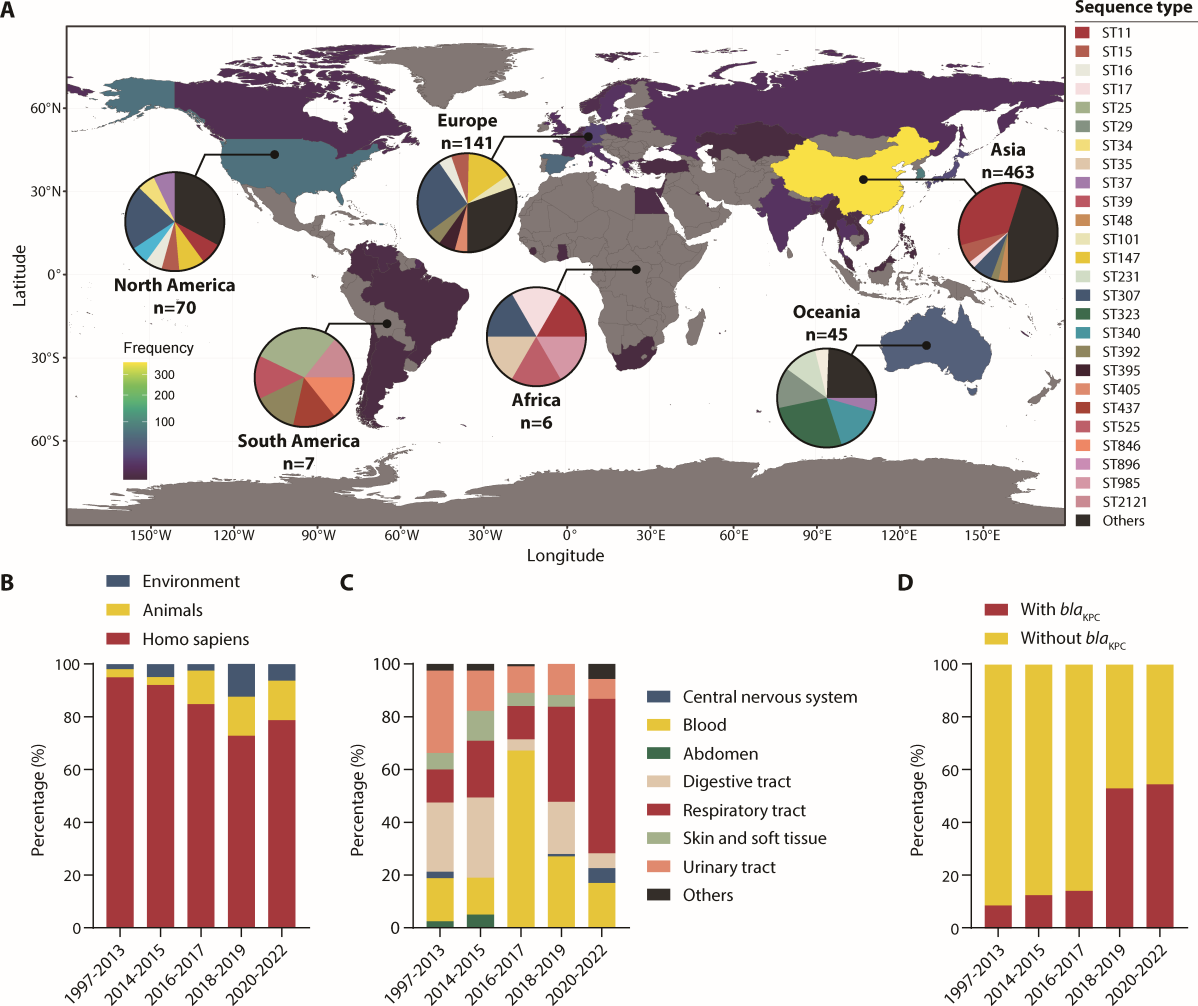
824 TPKPs from 1997 to 2022 were analyzed in this study, including their geographical distribution and STs (**A**), isolation sources (**B**), and infection sites (**C**). (**D**) Proportion of clinical TPKPs with or without *bla*_KPC_. STs of TPKPs were obtained by MLST and the map was generated with R and the ggplot2 package.

A systematic analysis of 824 *tetA*-positive *K. pneumoniae* (TPKP) genomes isolated from 1997 to 2022 revealed their distribution across 45 countries, with Asia harboring the highest prevalence (63.5%) and Africa the lowest (1.0%) (Fig. 1A). TPKPs exhibited striking genetic diversity, spanning 135 sequence types (STs), though ST11 dominated among Asian isolates (30.1%), while ST307 prevailed in Europe (22.9%) and North America (20.5%). Notably, while clinical sources accounted for >70% of isolates, environmental and animal-derived TPKPs surged post-2015 (7.0% to 28.1%), implicating non-human reservoirs as sources for emerging *tetA* dissemination (Fig. 1B). Clinically, TPKP infection sites shifted markedly over time: respiratory tract infections rose from 14.6% (1997–2013) to 55.4% (2020–2022), whereas urinary tract cases declined from 30.5% to 7.1% (Fig. 1C). Strikingly, the rate of clinical TPKPs co-harboring *bla*_KPC_ (KPC-TPKP) increased over fivefold from 9.7% (1997–2013) to 55.4% (2020–2022), with ST11 strains alone accounting for 58.5% (72/123) of all KPC-TPKP isolates. These findings suggest that *tetA* might have provided *bla*_KPC_-positive *K. pneumoniae* (KPC-KP) with selective advantages resisting tetracycline antibiotics, offering a potential explanation for the success of the highly transmissible ST11 lineage.

### Phylogenetic diversification and structural hotspots of TetA variants

**Fig 2:**
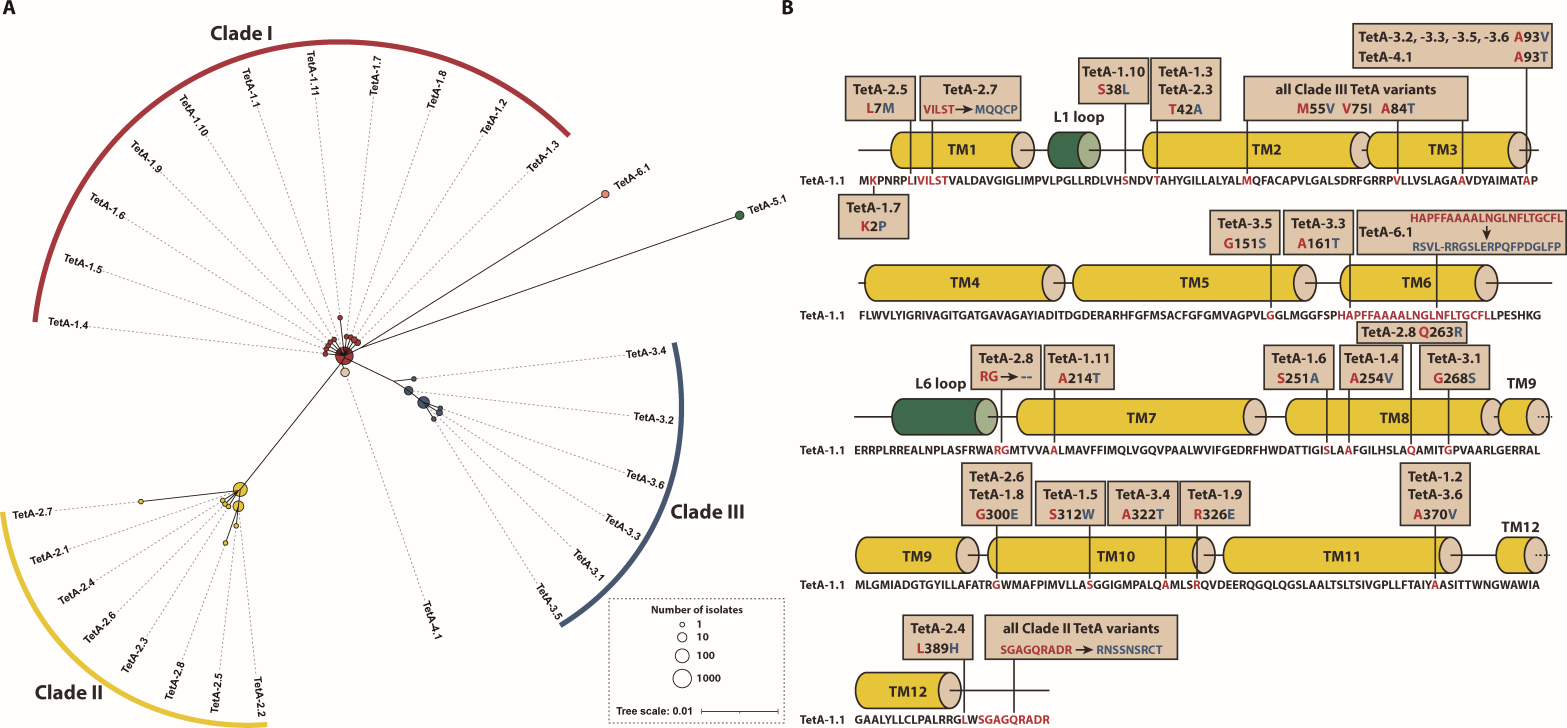
Phylogenetic tree (**A**) and the mutations of each TetA variant mapped onto their predicted secondary structure (**B**).

Classification of 28 TetA variants by prevalence and phylogeny resolved three Clades (I–III) with distinct evolutionary origins (Fig. 2A; Table S2), each of which was represented by an ancestral variant TetA-1.1, TetA-2.1, and TetA-3.1, respectively. The defining mutation that separates Clade II from Clade I is a C-terminal nine-amino acid substitution (SGAGQRADR→RNSSNSRCT), while the key mutation for distinguishing Clade III from Clade I is M55V/V75I/A84T/A93V/G268A. Strikingly, TetA-5.1—the most divergent variant—was found to be most frequently associated with *Enterobacter hormaechei* by BLASTn analysis (Fig. S1), implicating inter-genus horizontal transfer. Structural prediction of TetA-1.1 by AlphaFold3 localized mutation hotspots to transmembrane domains TM3, TM8, and TM10 (Fig. 2B), suggesting that these regions are critical for substrate recognition and efflux efficiency.

### Plasmid dynamics drive clade-specific TetA spread

**Fig 3:**
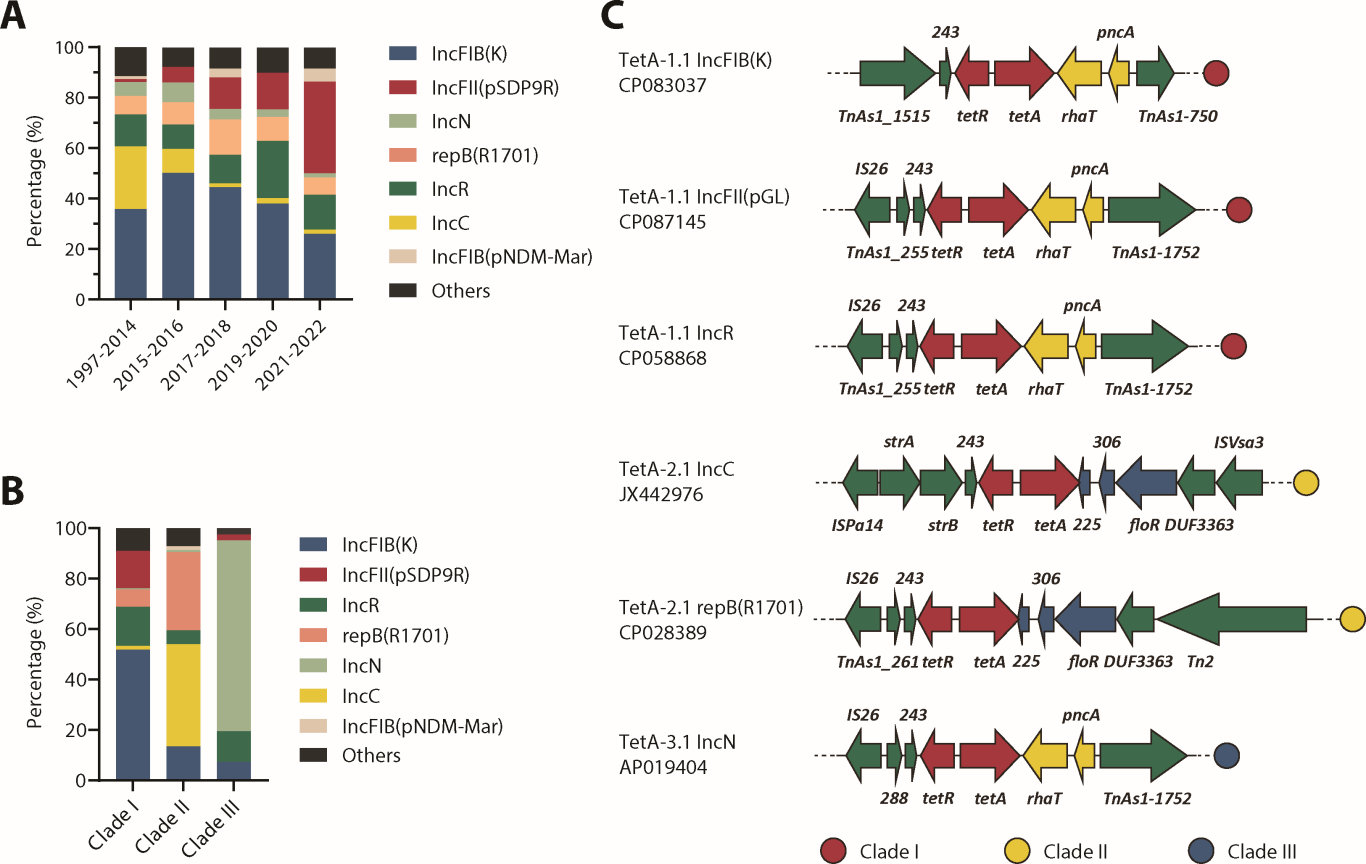
Shifting trends of replicon distribution of *tetA* plasmids over 1997–2022 (**A**). Replicon distribution (**B**) and the neighboring genes (**C**) of *tetA* representative of Clades I–III.

To gain insights into the horizontal transfer of TetA variants, we analyzed the genetic environments of *tetA* variants. TetA variants predominantly resided on IncFIB(K), IncFII(pSDP9R), and IncN plasmids: IncFII(pSDP9R) surged by 30-fold from 1.2% (1997–2014) to 36.2% (2021–2022), displacing IncC plasmids, which dropped from 24.8% to 1.7% in the same period (Fig. 3A). Clade-specific plasmid associations emerged: Clade I favored IncFIB(K) plus IncR plasmids (together 67.5%), Clade II IncC plus repB(R1701) (together 71.4%), and Clade III IncN (75.6%) (Fig. 3B). Genetic context analysis revealed conserved downstream elements: *pncA* (putative nicotinamidase-related amidase) and *rhaT* (putative drug/metabolite transporter superfamily permease) in Clades I/III vs *floR* (chloramphenicol efflux pump) and a 225-bp hypothetical gene-*hp225* (Fig. 3C). Notably, *hp225* has a 27-bp overlap with *tetA* in Clade II plasmids, responsible for Clade II’s defining C-terminal substitution, highlighting plasmid architecture as a driver of TetA diversification.

### TetA VWPRs: resistance gains and ecological costs

**Fig 4:**
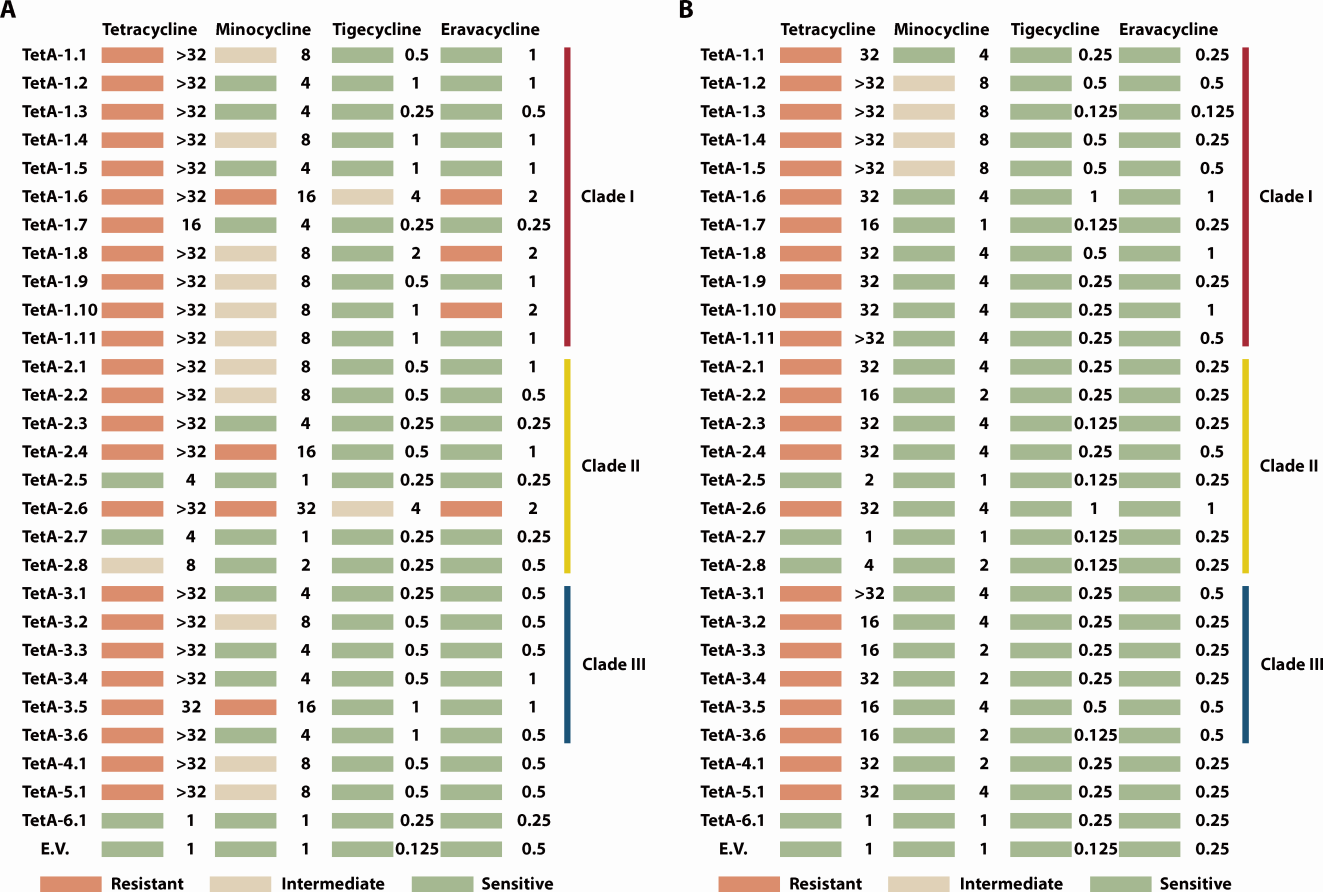
Resistance phenotypes and MICs of *K. pneumoniae* TU37 (**A**) and *E. coli* MG1655 (**B**) toward four generations of tetracycline antibiotics upon expression of *tetA* variants on a low-copy plasmid. Breakpoints for tigecycline: susceptible ≤2 mg/L, intermediate 4 mg/L, resistant ≥8 mg/L and for eravacycline: susceptible ≤1 mg/L, resistant >1 mg/L. E.V.: empty vector control.

To investigate the ability of the 28 TetA variants to mediate resistance to tetracycline family antibiotics, we measured the MICs of tetracycline family antibiotics after expressing *tetA* variants with *tetR* from their native genetic background on a low-copy plasmid in *K. pneumoniae* TU37 (ST23) and *E. coli* MG1655 (Fig. 4). Resistance phenotypes conferred by TetA variants decreased with the generational advancement of tetracycline antibiotics (Fig. 4). 85.7% of the variants confer resistance to tetracycline in both species, with TetA-2.5, TetA-2.7, TetA-2.8, and TetA-6.1 having MICs indistinguishable from the empty plasmid control, indicating that these variants might have lost their efflux ability due to their mutations. In contrast, four variants (TetA-1.6, TetA-2.4, TetA-2.6, and TetA-3.5) are capable of mediating resistance to the second-generation minocycline in *K. pneumoniae*. Notably, even to the third-generation tigecycline, TetA-1.1 led to fourfold higher MIC in *K. pneumoniae*. However, none of the 28 TetA variants mediated resistance above the clinical breakpoint (≥4 mg/L), with only TetA-1.6 and TetA-2.6 showing intermediate resistance in *K. pneumoniae*. Surprisingly, more variants (TetA-1.6, TetA-2.6, TetA-1.8, and TetA-1.10) conferred resistance against the fourth-generation eravacycline in *K. pneumoniae*, a novel phenotype for TetA. Based on the resistance phenotype to tigecycline and eravacycline, TetA-1.6, TetA-1.8, TetA-1.10, and TetA-2.6 were classified as VWPRs.

Paradoxically, despite conferring advanced tetracycline resistance, these VWPRs showed minimal epidemiological spread, with each variant only reported in patients once. This epidemiological paradox prompted us to investigate potential fitness costs through growth kinetic assays under nutrient-deprived (M9 minimal media) and weakly acidic (pH = 5.0 LB) conditions in two *K. pneumoniae* strains: TU37 (ST23) and HS11286 (ST11 CRKP). To our surprise, in HS11286, TetA-1.8 and TetA-1.10 conferred a significant growth advantage during exponential phase (5–12 hours) compared to TetA-1.1 in both LB and M9 media (Fig. S2A and C). Conversely, in TU37, TetA-1.6 expression caused a minor (≈5%) reduction in stationary-phase optical density in both normal and acidic LB, while showing no significant fitness defects in M9 (Fig. S2D and E).

**Fig 5:**
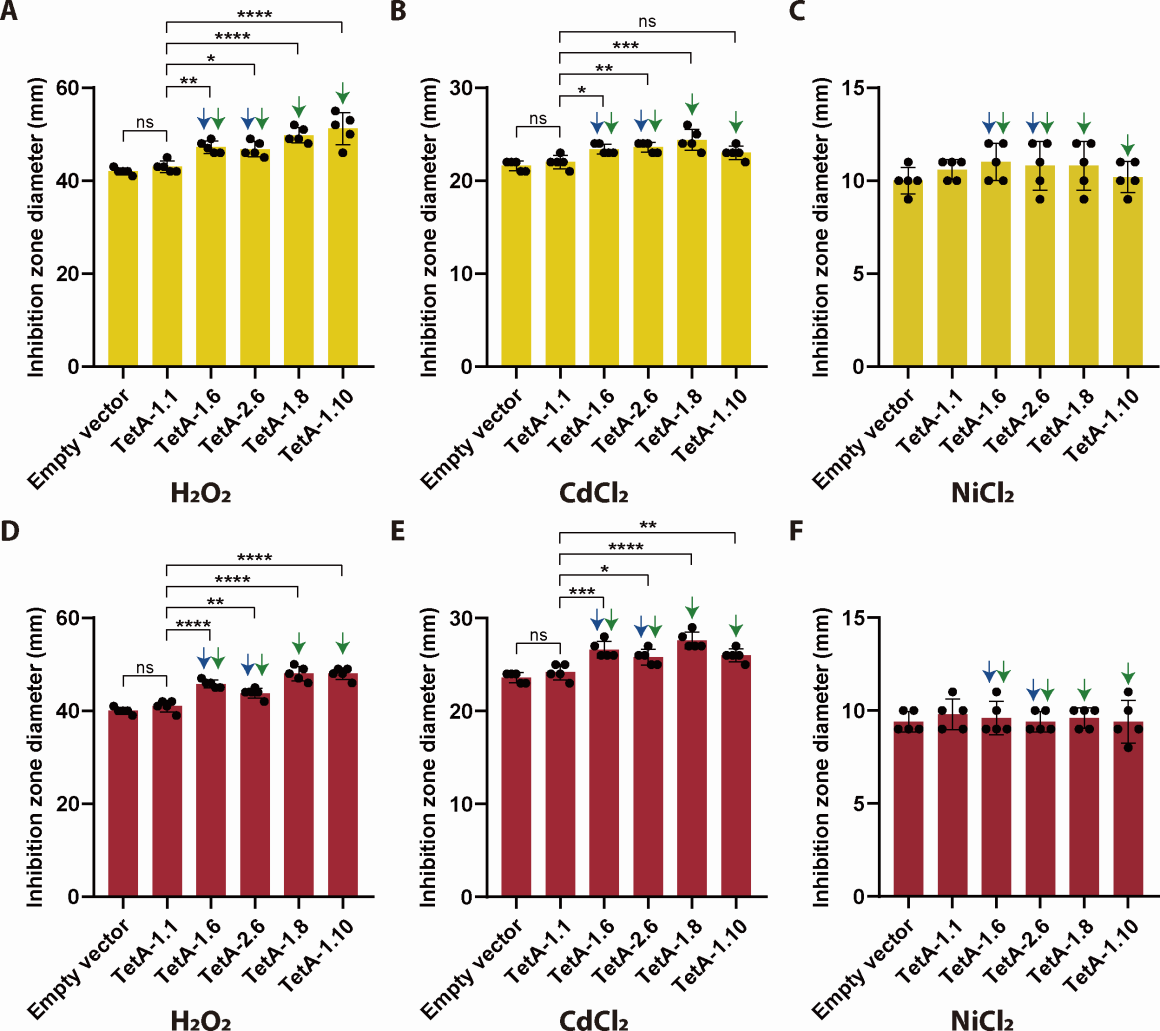
Inhibition zone diameters of *K. pneumoniae* TU37 (**A–C**) and HS11286 (**D–F**) expressing TetA variants to H_2_O_2_, CdCl_2_, and NiCl_2_. Significance thresholds: *P < 0.05, **P < 0.01, ***P < 0.001, ****P < 0.0001.

The observation that VWPRs exhibited minor growth defects despite clinical scarcity motivated us to systematically evaluate their collateral sensitivity to environmental stressors. We selected a panel of hospital-relevant disinfectants including H_2_O_2_, disinfectant “84” (main ingredient: HClO), and quaternary ammonium compounds alongside environmental heavy metals (CdCl_2_, NiCl_2_, K_2_TeO_3_, and CuSO_4_) using disc diffusion assays. To bypass TetR-mediated repression, tetracycline was included as an inducer in the initial screen. Notably, expression of TetA-1.6 conferred hypersusceptibility to NiCl_2_ and CdCl_2_ than TetA-1.1 in both *K. pneumoniae* strains, and expression of TetA-1.8/1.10 resulted in higher sensitivity to H_2_O_2_ (Fig. S3A and D), while VWPRs showed equivalent susceptibility to other disinfectants and heavy metals tested compared to TetA-1.1 (Fig. S4). To rule out the potential confounding effects of tetracycline’s antimicrobial activity, we repeated assays with H_2_O_2_, CdCl_2_, and NiCl_2_ using aTC—a tetracycline analog with minimal antibacterial activity—as the inducer. Indeed, both *K. pneumoniae* strains displayed significantly lower sensitivity to NiCl_2_ (>30%) and CdCl_2_ (>20%), while differences in susceptibility of NiCl_2_ between variants were no longer observed (Fig. 5B, C, E and F), suggesting that tetracycline may synergistically enhance metal toxicity. Surprisingly, this aTC-based assay exacerbated H_2_O_2_ and CdCl_2_ toxicity in VWPR-expressing strains (Fig. 5A, B, D and E).

As disinfectants could also be applied to already established bacterial reservoirs in various clinical or environmental scenarios, we sought to test if the expression of VWPRs modulates the tolerance of *K. pneumoniae* to H_2_O_2_ and CdCl_2_ by conducting time-kill assays. Notably, expression of TetA variants led to a significant reduction of tolerance to both H_2_O_2_ and CdCl_2_ compared to the empty vector control (Fig. S5A through C). While the increased tolerance of TetA-1.1-expressing strains compared to VWPR-expressing strains did not reach statistical significance (*P* = 0.08–0.14 at 2 h post H_2_O_2_ treatment) due to time-kill assay variability, a consistent numerical trend was observed for both stressors (Fig. S5A through D). Overall, our findings underscore ecological trade-offs that constrain VWPRs to transient clinical niches rather than environmental persistence.

## DISCUSSION

Since the initial linkage of *tetA* to tigecycline resistance in *K. pneumoniae* (17), a few *tetA* variants have been shown to mediate tigecycline resistance in *K. pneumoniae* (27, 28). Moreover, an epidemiological description of *tetA* in *K. pneumoniae* is still lacking. Our global analysis revealed that the association of *tetA* with multidrug-resistant high-risk clones—ST11 in Asia (29), ST307/ST147 in Europe/North America (30), and ST323 in Australia (31)—has become stronger, placing *tetA* as a novel genomic hallmark of these epidemic lineages, raising concerns that TetA-mediated resistance to advanced tetracyclines (e.g., eravacycline) could undermine last-line therapies. Unlike other clinically important resistance genes such as *bla*_KPC_ and *mcr*, whose variants and resistance phenotypes have been well characterized (32, 33), TetA variants remain systematically underexplored. To fill this gap, we searched the NCBI database and obtained 824 TPKPs from 1997 to 2022, from which we identified 28 variants and classified them into 3 major clades. TetA-1.1 was initially reported as *tet*(A) type I variant (Tet(A)v1) (17). Thus, we believe it is more appropriate to designate *tetA-1.1* as the “wild-type” *tetA* allele in *K. pneumoniae* due to its prevalence (73.7%). Moreover, *tetA* variants exhibit clade-specific plasmid associations. Given the crucial roles played by plasmids in resistance transmission (34, 35), our findings enable real-time tracking of variant evolution, mechanistic dissections of the *tetA* transmission dynamics, and the potential design of novel plasmid-centric interventions.

Eravacycline, a synthetic fluorocycline, is a fourth-generation tetracycline (36). Since its approval by the FDA in 2018, reports of eravacycline resistance in *K. pneumoniae* have remained scarce. The identification of TetA-1.6/1.8/1.10/2.6 as eravacycline resistance determinants expands the known mechanisms of fluorocycline resistance beyond TetX enzymes and efflux pumps such as OqxAB (8, 12), highlighting the underappreciated role of TetA variants in shaping resistance. Strikingly, both TetA-1.6 (S251A) and TetA-1.8 (G300E) emerged during tigecycline therapy (16, 37, 38), suggesting that sequential exposure to antibiotics selects for “pan-tetracycline” MFS transporters. In contrast, TetA-2.6 (G300E + C-terminal SGAGQRADR to RNSSNSRCT) and TetA-1.10 (S38L) were both recovered from patients (39) and emerged in the absence of tigecycline treatment, hinting at the presence of stealthy evolutionary pathway without direct selection. Notably, TetA-2.6 exhibited higher resistance to minocycline and tigecycline than TetA-1.8, suggesting that functional divergence served as the basis for clade formation. The identification of these TetA variants not only expands the mechanistic repertoire of eravacycline resistance but also underscores TetA as an evolutionarily adaptable and clinically underappreciated resistance determinant, demanding prioritized surveillance.

*K. pneumoniae* is known to be a key trafficker of AMR genes from environmental microbes to clinically important pathogens (19, 40). Here, this is exemplified by the spread of TetA to 135 distinct STs and its continued isolation from animals and environmental sources (41, 42). Despite their clinical emergence, VWPRs so far have demonstrated restricted dissemination, a paradox reflected by ecological trade-offs. In this study, strains expressing all four VWPRs exhibited heightened susceptibility to H_2_O_2_ (which is ubiquitous in healthcare settings) and the heavy metal cadmium. We employed two complementary methodologies to assess resistance and tolerance, each with distinct advantages and limitations. First, the disc diffusion assay, a well-established and reproducible technique for resistance profiling, excels in detecting subtle differences between strains as it generates a continuous gradient of stressors tested. However, this method remains largely qualitative. Second, the time-kill assay provides quantitative insights into tolerance but exhibits inherent variability in kinetic measurements, which may affect reproducibility.

Our findings are consistent with recent evidence that TetA-1.1 imposes fitness costs for growth (37), with the concept now extended to trade-offs for susceptibility to environmental factors. Heavy metals exert significant selective pressures on bacteria in the environment, which is evidenced by the ubiquitous carriage of heavy metal resistance genes (HMRGs) by multidrug-resistant *Enterobacteriaceae*, such as *K. pneumoniae* (43, 44). Thus, heavy metals enriched in agricultural/industrial habitats by anthropogenic activities (45) likely purge TetA VWPRs from environmental reservoirs, restricting their success. Thus, while clinical antibiotic pressure selects for TetA diversification, environmental metal stress counter-selects against the spread of VWPRs—a delicate equilibrium exploitable for intervention.

In summary, our findings suggest that dissemination of resistance could be viewed as an ecological tug-of-war, in which the stability of VWPRs is kept in a delicate equilibrium; the benefits of antibiotic resistance are offset by collateral sensitivity to other agents. This such informs approaches for a sustainable One Health strategy to mitigate dissemination of resistance to advanced tetracyclines, which integrates four different dimensions: (i) antibiotic stewardship to reduce eravacycline/tigecycline selection, (ii) application of H_2_O_2_-based disinfectants and monitor resistant mutants, (iii) sampling of heavy metal polluted hotspots to watch for acquisition events of HMRGs by TPKPs, and (iv) plasmid-centric surveillance prioritizing the major replicons.

## SUPPLEMENTAL MATERIAL

Supplemental figures aac.00671-25-s0001.docx Fig. S1 to S5.

Supplemental tables aac.00671-25-s0002.xlsx Tables S1 to S5.

ASM does not own the copyrights to Supplemental Material that may be linked to, or accessed through, an article. The authors have granted ASM a non-exclusive, world-wide license to publish the Supplemental Material files. Please contact the corresponding author directly for reuse.
