# Supplementary material for: Phylogenetic diversification and fitness trade-offs of TetA variants in mediating eravacycline resistance in Klebsiella pneumoniae
Source: Antimicrob Agents Chemother. 2025 Dec 30;70(2):e00671-25. doi: 10.1128/aac.00671-25 (PMC12888893; doi:10.1128/aac.00671-25)
Supplement: Supplemental figures — Fig. S1 to S5. [file aac.00671-25-s0001.docx]

**Supplementary materials**

**
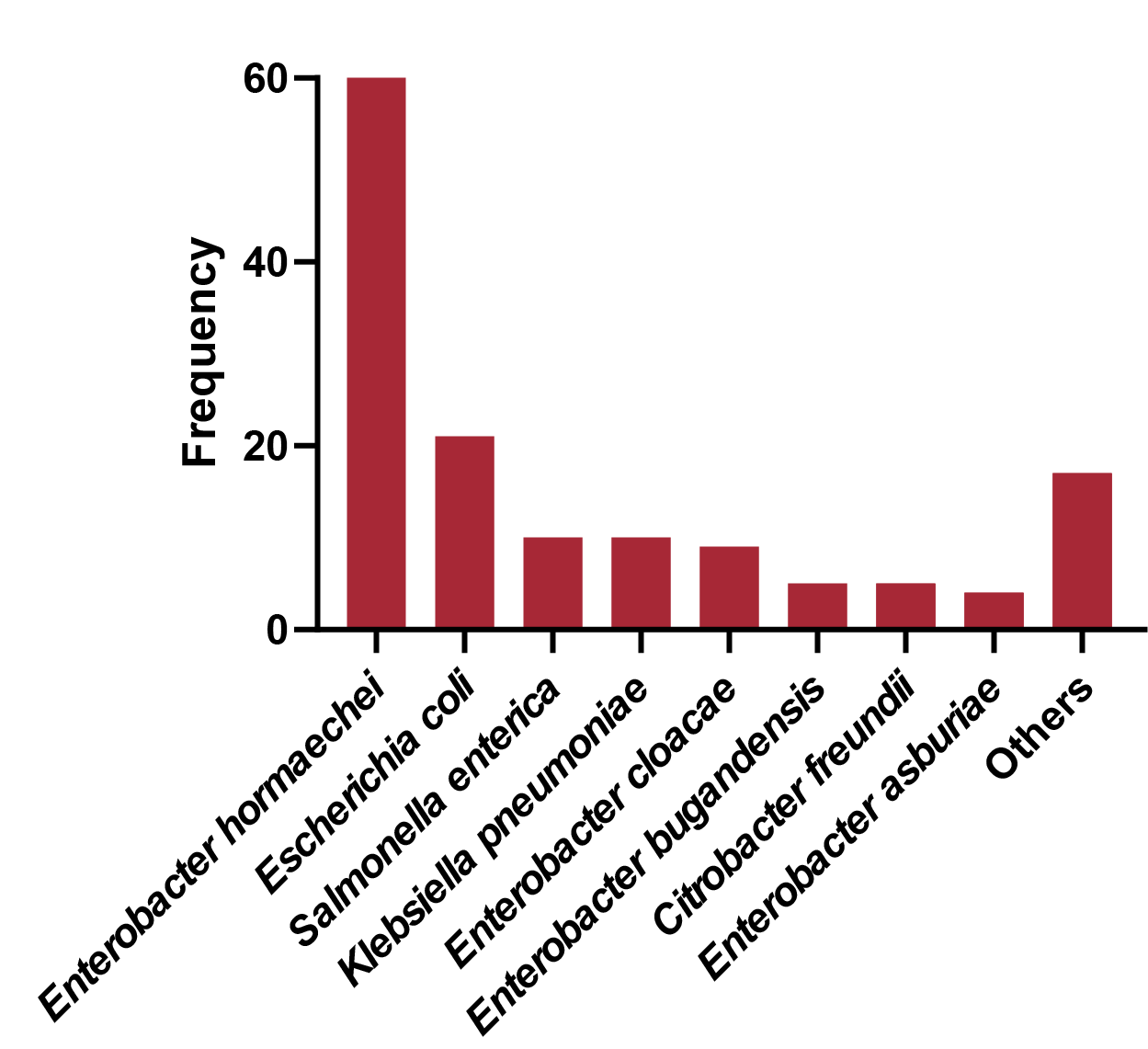
**

**Fig S1.** Frequency distribution of *tetA-5.1* gene among all bacterial species analyzed by BLASTn.

**
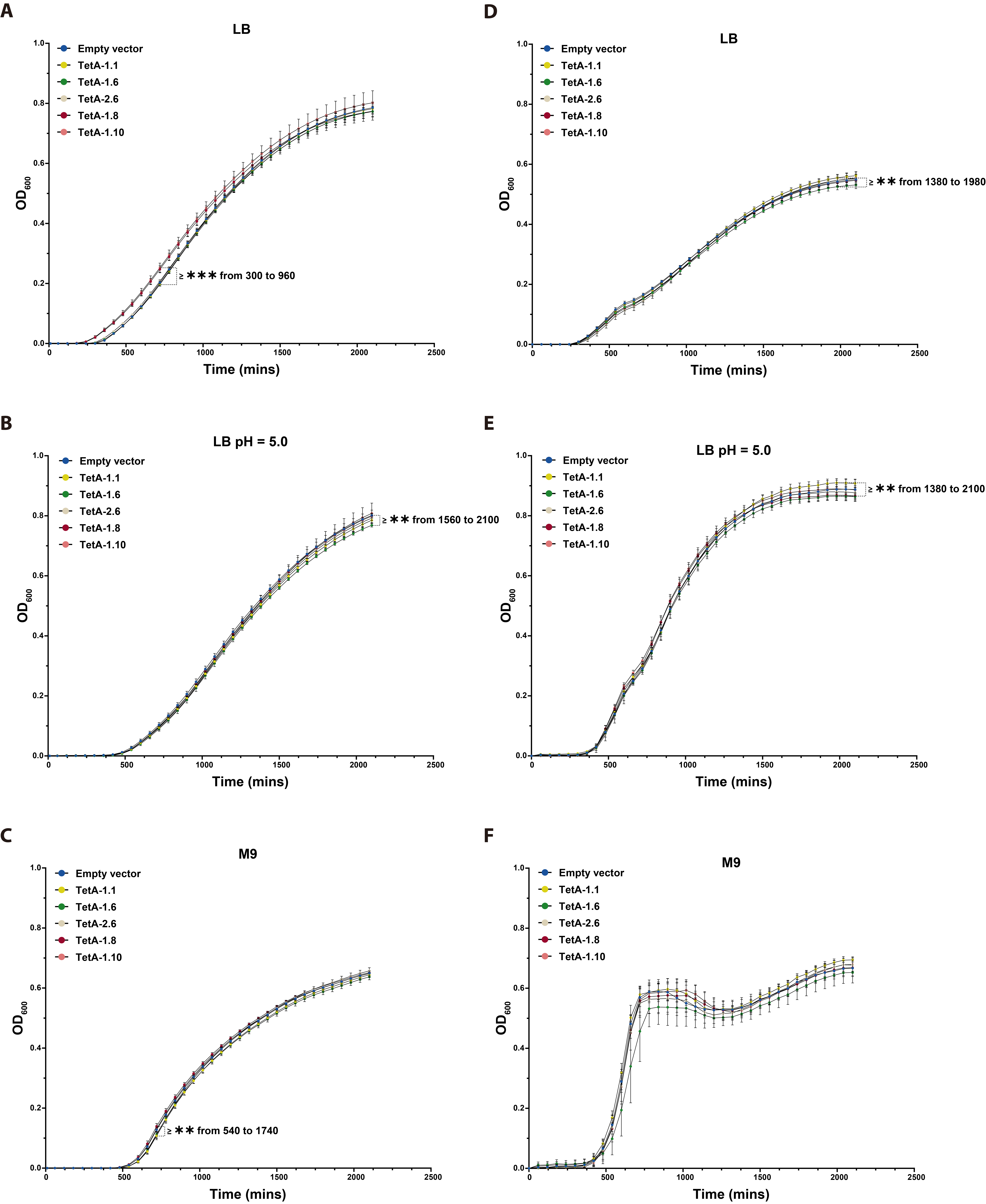
**

**Fig S2.** Growth of *K. pneumoniae* HS11286 (A-C) and TU37 (D-F) expressing TetA variants in LB, acidic LB (pH = 5.0), and M9. Significance thresholds: **P < 0.01, ***P < 0.001. Inducer: anhydrotetracycline.


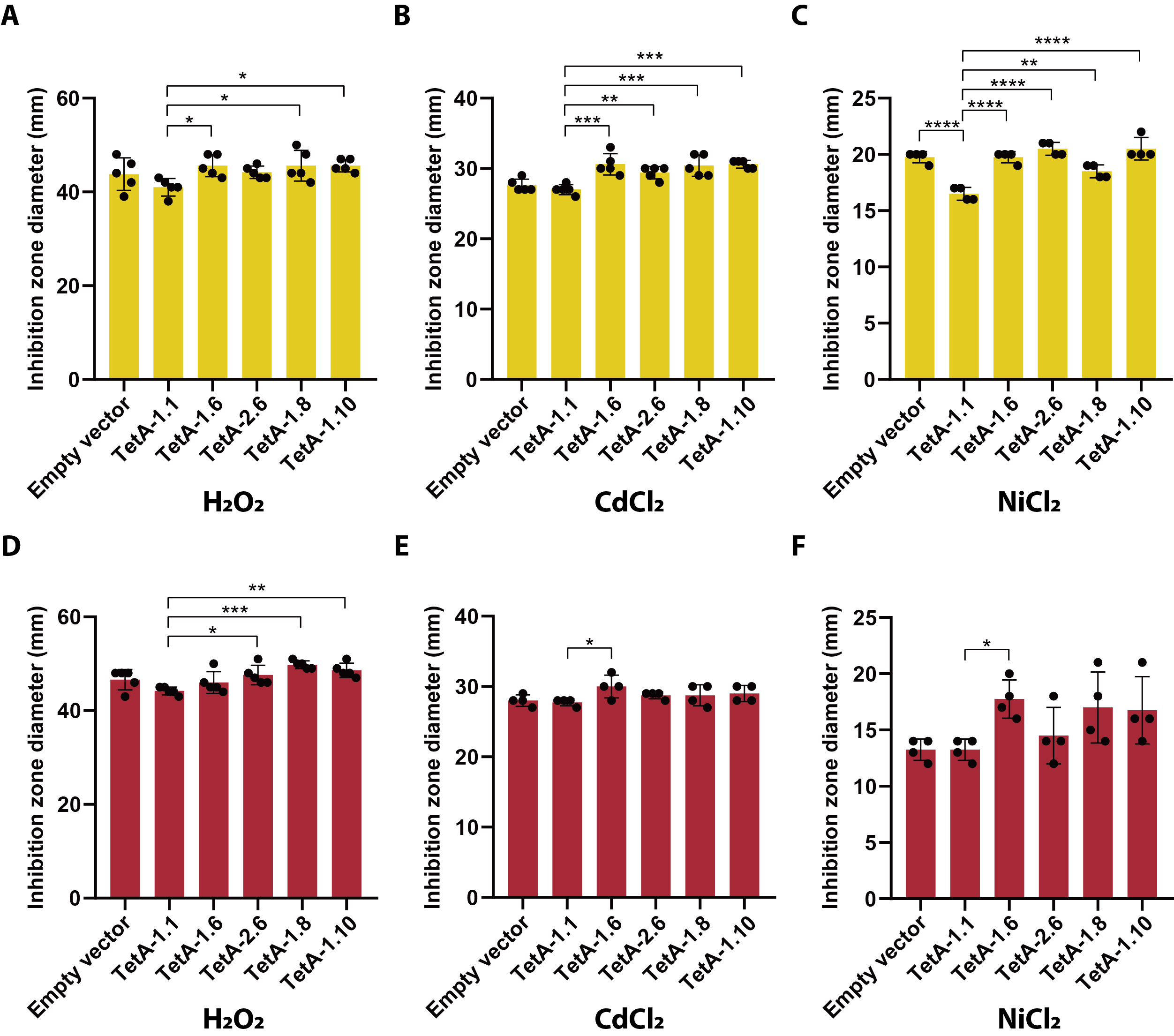


**Fig S3.** Inhibition zone diameters of *K. pneumoniae* TU37 (A-C) and HS11286 (D-F) expressing TetA variants to H_2_O_2_, CdCl_2_, and NiCl_2_. Inducer: tetracycline. Significance thresholds: *P < 0.05, **P < 0.01, ***P < 0.001, ****P < 0.0001.

**
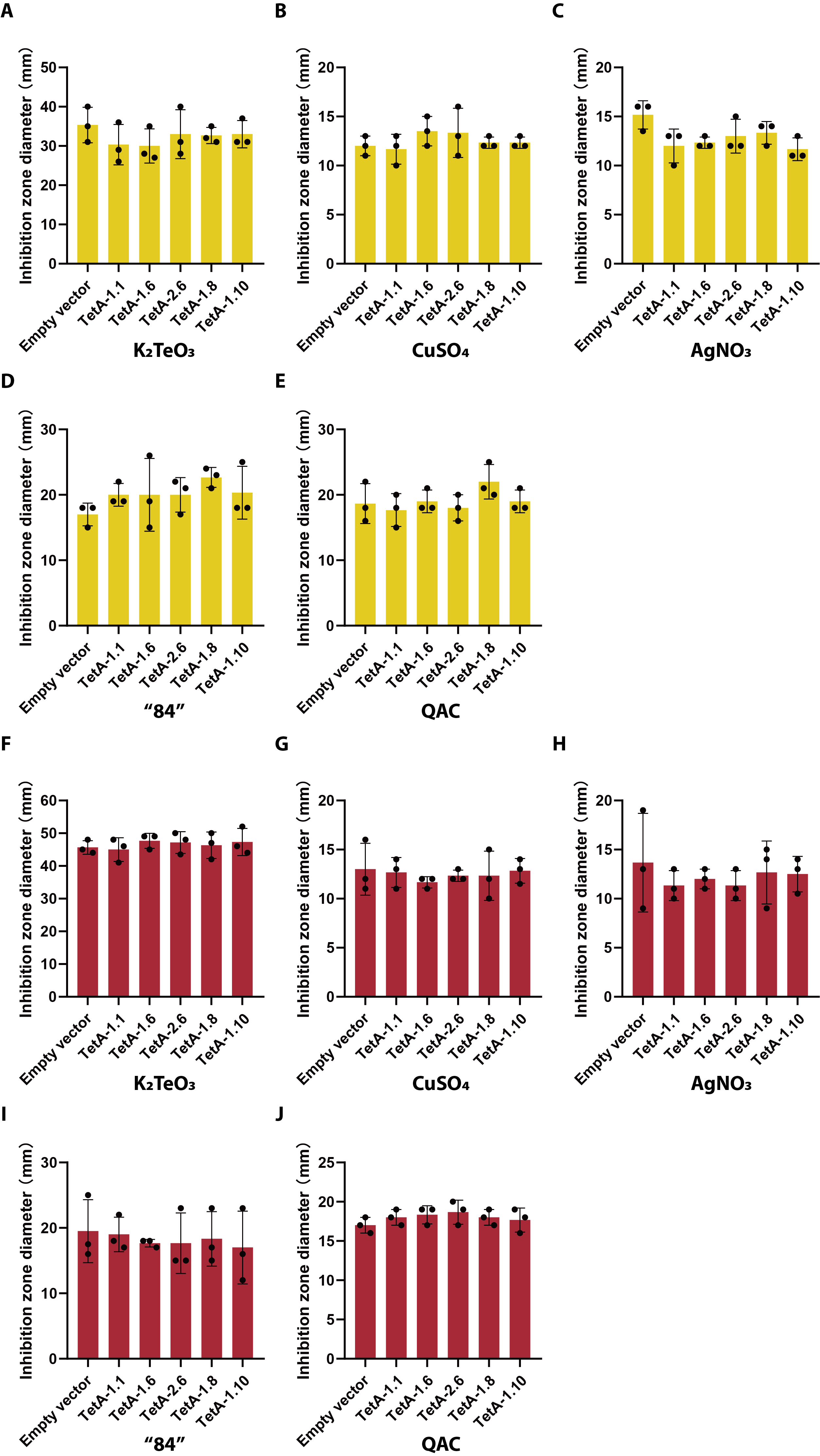
**

**Fig S4.** Inhibition zone diameters of *K. pneumoniae* TU37 (A-E) and HS11286 (F-J) expressing TetA variants to K_2_TeO_3_, CuSO_4_, AgNO_3_, disinfectant “84”, and QACs. Inducer: tetracycline.


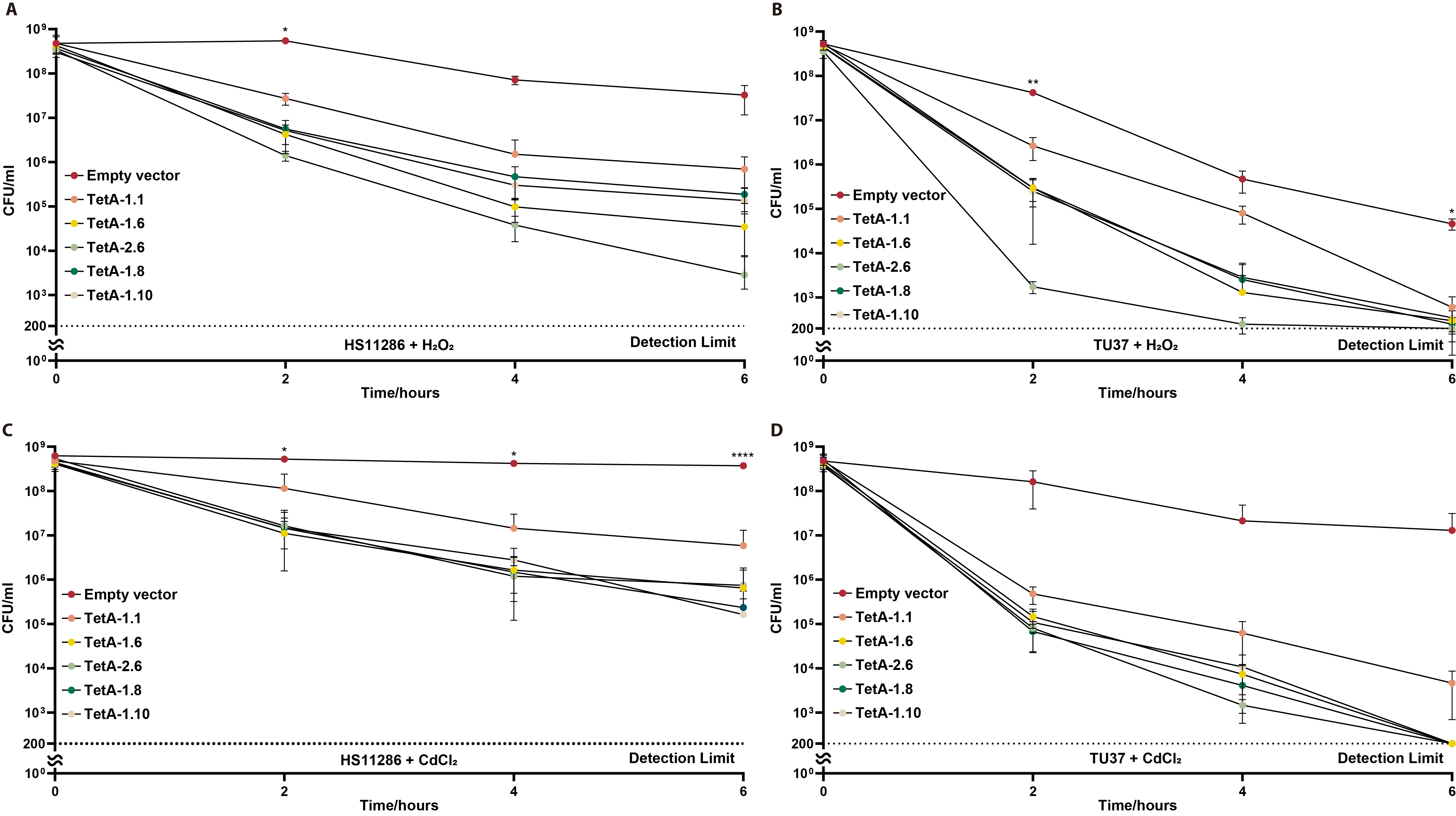


**Fig S5.** Number of viable *K. pneumoniae* expressing TetA variants over time after being challenged with 0.012% H_2_O_2_ (v/v) or CdCl_2_ (40 mM for TU37, 2.5 mM for HS11286). Inducer: anhydrotetracycline. Significance thresholds: *P < 0.05, **P < 0.01, ****P < 0.0001.
